# Four conservation challenges and a synthesis

**DOI:** 10.1002/ece3.10052

**Published:** 2023-05-02

**Authors:** Byron K. Williams, Eleanor D. Brown

**Affiliations:** ^1^ Science and Decisions Center U.S. Geological Survey Reston Virginia USA

**Keywords:** conservation, environmental variation, nonstationarity, partial observability, structural uncertainty, system dynamics

## Abstract

Conservation and management of biological systems involves decision‐making over time, with a generic goal of sustaining systems and their capacity to function in the future. We address four persistent and difficult conservation challenges: (1) prediction of future consequences of management, (2) uncertainty about the system's structure, (3) inability to observe ecological systems fully, and (4) nonstationary system dynamics. We describe these challenges in terms of dynamic systems subject to different sources of uncertainty, and we present a basic Markovian framework that can encompass approaches to all four challenges. Finding optimal conservation strategies for each challenge requires issue‐specific structural features, including adaptations of state transition models, uncertainty metrics, valuation of accumulated returns, and solution methods. Strategy valuation exhibits not only some remarkable similarities among approaches but also some important operational differences. Technical linkages among the models highlight synergies in solution approaches, as well as possibilities for combining them in particular conservation problems. As methodology and computing software advance, such an integrated conservation framework offers the potential to improve conservation outcomes with strategies to allocate management resources efficiently and avoid negative consequences.

## Graphical abstract

Four ubiquitous challenges to effective conservation are as follows: prediction of the future impacts of decisions; inability to observe biological systems fully; uncertainty about system structure; and nonstationary system dynamics. We present a basic Markovian framework, encompassing approaches to all four challenges, which features technical linkages among the challenges and thus allows synergies in solution approaches for particular conservation problems. The framework offers the potential to improve conservation outcomes with strategies that allocate management resources more efficiently and avoid negative consequences.

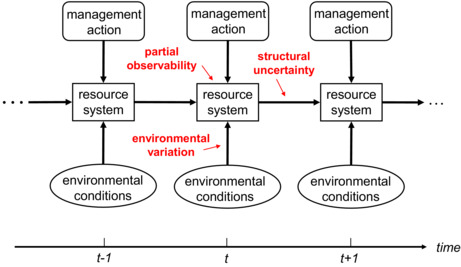

## 1 INTRODUCTION

Biological conservation tends to be both dynamic and uncertain. It is dynamic in that both the protection of extant ecological systems and maintenance of the ecological processes that sustain them play out over the long term (Pressey et al., 2007; Sarkar et al., 2006). It is uncertain in that ecological systems are inherently random, and any understanding of system conditions and dynamics is always incomplete (Williams et al., 2002). The effectiveness of conservation often depends on how well system dynamics and their associated uncertainties are represented in conservation planning and decision‐making.

Formal decision‐making in ecology can take many forms, depending on the conservation problem at hand. Modeling frameworks might include, for example, continuous or discrete system states; lags in state dynamics and delays in policy implementation; hysteresis in system dynamics; deep uncertainty about system states; discontinuous environmental and management effects; and other features. Here we represent system behaviors with Markovian transitions with discrete states and actions over a countable time horizon, as an effective way to assess and compare the problems considered in this paper. Though necessarily restricted in its range of applicability, such a framework is broadly useful for representing ecological systems and widely adopted in conservation. With it we can consider sources of uncertainty that have long hindered the effectiveness of conservation and clarify some important relationships in treatments of them.

Uncertainties about the structure of ecological systems and the impacts of environmental conditions and management activities create difficulties in virtually all conservation efforts, regardless of geographic scale and ecological context. In this paper we address challenges in accounting for future impacts of management and for uncertainties about the structure, status, and dynamics of biological systems. We focus specifically on four ubiquitous conservation challenges, namely (1) prediction of future consequences of management, (2) uncertainty about the system's structure, (3) inability to observe ecological systems fully, and (4) nonstationary system dynamics. The overall issue can be considered to be one of predictability, given an inevitable lack of precision in predicting the future no matter how well one understands and models and observes a system. Thus, the first‐listed challenge addresses variation in system behaviors in the absence of other uncertainties such as structural uncertainty and partial observability, and each of the other challenges includes an additional uncertainty factor in the predictive model.

We discuss how all four challenges can be addressed within a basic framework of Markovian transitions subject to different sources of uncertainty. Markovian transitions with discrete states and actions operating over a countable time horizon can be applied to each challenge. Each requires specific variations of the basic framework, including adaptations of state transition models, methods for tracking uncertainty, and value assessments.

## 2 CONSERVATION ARCHITECTURE

We describe conservation here in terms of a dynamic natural system that changes over an indefinite time horizon in response to fluctuating environmental conditions and management actions. Demographic stochasticities and random environmental variation induce unpredictability in system behaviors, and management actions affect transitions in terms of immediate change, as in altering system states such as population size, and ongoing change, as in influencing ecological processes or vital rates that drive system change. The goal is to select actions over time that are likely to produce the greatest conservation benefit in the long term.

Key elements of our conservation architecture include:

**Time horizon**: Ecological dynamics can be thought of in terms of a time horizon that may be discrete or continuous, commencing at some initial time, say $t_{0}$, and terminating at time *T*. We consider discrete time horizons that are delineated in unit increments of time starting at $t_{0}=0$. Thus, ecological status and change are recorded at times $t\in \left\{0,1,2,\ldots ,T\right\}$, where *T* is potentially unspecified and may be infinite.

**System states**: Ecological systems of organisms interacting with their habitats in a dynamic environment can be represented in terms of system states and trajectories of change over time. System state is assumed to vary in response to fluctuating conditions and management actions. Examples might include population size or density, population vital rate, spatial distribution, or biological diversity. For convenience, we assume that the system state can be represented in terms of discrete states over an indefinite time horizon.

**Management actions**: Iterative decision‐making involves choosing actions at multiple decision points over time. Action taken at a particular time produces immediate returns and influences future action by affecting transitions to future states over the remainder of the timeframe. Actions might include species introductions, habitat manipulation, contaminant clean‐up, or enforcement of regulatory rules. A key issue is to identify a useful set of potential actions from which to choose a specific action at each decision point. If available alternatives are too limited, potentially important system responses may not be considered. Conversely, evaluation becomes more difficult as the range of alternatives expands (Williams & Brown, 2016).

**State transitions**: Effective decisions can be identified by comparing alternative actions in terms of immediate returns and future consequences. Transition models linking actions to system consequences facilitate the comparison of strategies and models, by forecasting system changes and highlighting uncertainty about system structure and function. It must be possible to identify models that adequately represent system dynamics; otherwise, comparisons of models' responses with actual data are unlikely to produce useful conservation strategies (Runge & Johnson, 2002).

**Status tracking**: Tracking the system's status and evaluating management performance with the resulting data provide a means by which to identify best management, learn about system dynamics, and meet management goals. Tracking is useful to the extent that its focus is based on the larger management context of which it is a part, and it should be designed accordingly (Nichols & Williams, 2006).

**Immediate returns and costs**: Actions taken at particular times may not only produce immediate returns but also incur associated costs. For example, translocation of organisms into an area leads to an immediate change in population status, but at the expense of associated labor and equipment costs. The accumulation of such benefits and costs provides a metric for evaluating actions over time.

**Valuation**: Conservation is guided by value functions that help to determine management effectiveness. Valuation typically aggregates discounted returns or costs, or both, over time, and comparison of value across management strategies allows one to search among different strategies for positive conservation results and limited losses. Because valuation is tied to the structure of a value function and the magnitude of discounting, it is important to choose value components carefully.

## 3 DYNAMIC APPROACH TO THE FOUR CONSERVATION CHALLENGES

We approach solutions to the ecological challenges mentioned in Section 1 within a framework of Markovian transitions involving different sources of uncertainty. Transitions among states are assumed to be controlled by time‐specific actions, tracked by monitoring, and evaluated over time. We use aggregated returns (possibly net of costs) as a basis for selecting and evaluating management strategies. With this framework, we discuss structural uncertainty, imperfect observability of states, nonstationary system change, and future consequences of present actions.

### 3.1 Dealing with the future

A fundamental challenge in ecological management is to balance immediate returns from actions against future ecological status and returns, given that current action can influence present as well as future status. In many applications, conservation practice focuses on immediate or equilibrium conditions, with little regard for the trajectory of ecological change. An extreme example is the overexploitation of a biological population, as with heavy poaching of megafauna, excessive overfishing of fish stocks, or overgrazing on rangelands, which may maximize immediate yield but limit the capacity for regeneration. Managing in such a way as to trade off immediate and future returns is a significant challenge with ecological systems. Even a cursory look at the ecological record reveals a propensity to talk about the future, but little follow‐up that includes the design and implementation of conservation strategies over time (Nichols & Williams, 2012).

An approach to decision‐making that accounts for the future consequences of present management actions involves dynamic optimization, in the spirit of dynamic programming (Bellman, 1957; Marescot et al., 2013; Williams et al., 2002). One of the earliest and best examples in ecology was Anderson's (1975) treatment of the sport harvest of North American waterfowl. Since then, iterative decision‐making to optimize performance has become well established (Marescot et al., 2013; Williams et al., 2002). However, applications to specific ecological problems remain limited, in part because the approach is seen as technically complicated and its computing burden increases exponentially with the scale of the problem (Taylor, 1993).

Dynamic decision‐making in ecology uses models with transitions between successive system states, based on biological processes such as mortality, reproduction, and movement. We assume that transitions are Markovian, in that they are influenced at any time only by the current state and management action taken at that time, but not by previous history (Ross, 1996). Management of observable Markovian systems is comprehensively discussed in the optimal control literature (Bertsekas, 2012, 2017; Puterman, 1994).

**FIGURE 1:**
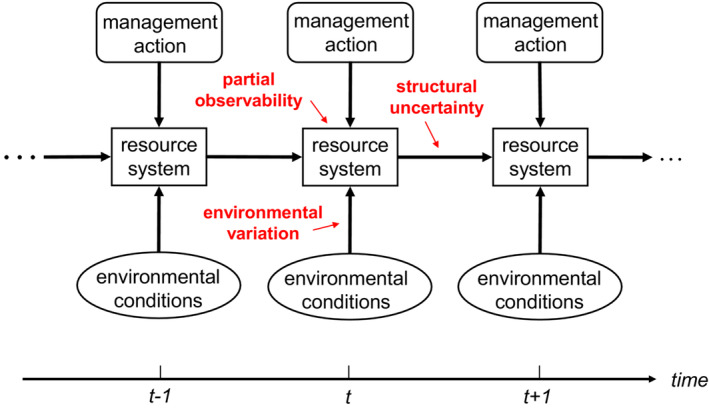
Ecological changes in response to management actions and environmental conditions. Fluctuating environmental conditions influence system dynamics, and management actions produce immediate returns and influence the trajectory of system changes. Uncertainty features include environmental variation, uncertainty about system structure and processes, and an inability to observe systems fully. After Williams and Brown (2016).

We focus here on ecological systems that change randomly over a discrete time horizon in response to fluctuating environmental conditions and time‐specific management actions (Figure 1). The system state at a particular time is represented by *x*, with management action *a* at that time and $x^{\prime }$ the subsequent state. System dynamics are described by single‐step transitions $x^{\prime }=F\left(x,a,z\right)$, where action *a* is one of a sequence of actions over the time horizon and *z* represents environmental variation. Demographic stochasticities and randomness in *z* induce Markovian transition probabilities $P\left(x^{\prime }|x,a\right)$ over the timeframe.

Policies for this problem consist of state‐specific actions, in which an action is specified for each state at each time over the defined time horizon. In mathematical terms, policy π specifies an action for each combination $\left(t,x\right)$, that is, $\pi_{t}\left(x\right)=a_{t}$. A special case involves stationary policies, in which the same action is identified for a given state irrespective of the time the state occurs, that is, $\pi_{t}\left(x\right)=\pi \left(x\right)=a$.

Valuation for this problem is tied to policies of state‐specific actions. A value function for a given policy aggregates discounted returns over an indefinite time horizon,

$$V_{\pi }\left(x\right)=E\left[\sum_{\tau =t}^{\infty }\lambda^{\tau -t}R\left(a_{\tau }|x_{\tau }\right)|x\right],$$

where the expectation accounts for randomness in process transitions through time. Value can be written recursively in terms of current and future expected returns,

(1) $$V_{\pi }\left(x\right)=R\left(a|x\right)+\lambda \sum_{x^{\prime }}P\left(x^{\prime }|x,a\right)V_{\pi^{\prime }}\left(x^{\prime }\right),$$

with policy expressed iteratively as $\pi =\left\{a,\pi^{\prime }\right\}$ (see Williams, 2009).

An elegant approach to dynamic decision‐making uses Bellman's equation (Bellman, 1957) to identify a stationary policy $\pi =\left\{a,\pi^{\prime }\right\}$ that optimizes $V_{\pi }\left(x\right)$ in Equation (1) (Derman, 1970; Puterman, 1994). Consider, for example, valuation at the terminus *T* of a finite time horizon. In this case, optimal actions are needed for only the terminal time, and they are found by simply optimizing over available actions for each state, that is,

$$\begin{array}{c} V\left[x\right]=\max_{a}\;R\left(a|x\right)\\ \;\;\;\;=R\left(a^{*}|x\right). \end{array}$$

With the last two times *T* − 1 and *T*, the problem involves the optimal selection of an action at *T* − 1, given that optimal values for states at time *T* are already known:

$$V\left[x\right]=\max_{a}\left\{R\left(a|x\right)+\lambda \sum_{x^{\prime }}P\left(x^{\prime }|x,a\right)R\left(a^{*}|x^{\prime }\right)\right\}.$$

By induction, a two‐step optimization of $V_{\pi }\left(x\right)$ involves maximizing the future over all policies conditional on an immediate action and then maximizing the current action given an optimal future:
(2)

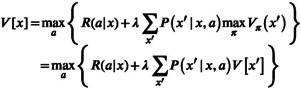

(Marescot et al., 2013: Table 1). An optimal policy consists of the state‐specific optimal actions

$$a^{*}=\underset{a}{\arg\max}\left\{R\left(a|x\right)+\lambda \sum_{x^{\prime }}P\left(x^{\prime }|x,a\right)V\left[x^{\prime }\right]\right\}$$

identified in the value optimization.

**TABLE 1:** Some challenges, uncertainties, and optimal value functions in conservation management.

Challenge	Uncertainty factors	Observability	Process structure	Optimal value function
Prediction of future impacts	Environmental variation	Full	Known	$V\left[x\right]=\max_{a}\left\{r\left(a|x\right)+\lambda \sum_{x^{\prime }}P\left(x^{\prime }|x,a\right)V\left[x^{\prime }\right]\right\}$
Uncertainty about structure	Environmental variation, structural uncertainty	Full	Not known	$V\left[x,q\right]=\max_{a}\left\{r\left(a|x,q\right)+\lambda \sum_{x^{\prime }}P\left(x^{\prime }|x,a,q\right)V\left[x^{\prime },q^{\prime }\right]\right\}$
Limited observability	Environmental variation, partial observability	Partial	Known	$V\left[b\right]=\max_{a}\left\{r\left(a|b\right)+\lambda \sum_{o^{\prime }}P\left(o^{\prime }|b,a\right)V\left[b^{\prime }\right]\right\}$
Mixture of limited and full observability	Environmental variation, partial observability	Full and partial	Known	$V\left[x,b_{y}\right]=\max_{a}\left\{r\left(a,|,x,,,b_{y}\right)+\lambda \sum_{x^{\prime }}P\left(x^{\prime }|x,a,b_{y}\right)V\left[x^{\prime },b_{y}^{\prime }\right]\right\}$
Nonstationary system	Environmental variation, structural uncertainty, time change in structure	Full	Not known	$V\left[x,q_{y}\right]=\max_{a}\left\{r\left(a,|,x,,,q_{y}\right)+\lambda \sum_{x^{\prime }}P\left(x^{\prime }|x,a,q_{y^{\prime }}\right)V\left[x^{\prime },q_{y^{\prime }}^{\prime }\right]\right\}$

By incorporating Markovian transitions $P\left(x^{\prime }|x,a\right)$ directly into decision‐making, a solution accounts for the future consequences of immediate actions. The optimal action identified in Equation (2) is seen to maximize the sum of current and best future returns, where subsequent valuations are conditional on the action taken. It is the linkage of prior action and posterior value that projects the influence of actions into the future. Accounting for the future in this way can involve a considerable computing cost (Marescot et al., 2013). However, advances in finding efficient solution approaches to dynamic decision problems (Wang et al., 2009) have extended the range of applicability in ecology and greatly increased our ability to identify effective strategies for long‐term conservation.

### 3.2 Dealing with structural uncertainty

Assessment of present and future conservation status and returns, as described in Section 3.1, is challenged by uncertainty about the system's structure and processes and the influence of management on them. Ecological relationships tend to be especially complex and subject to uncertainty, and any transition model can be seen as “wrong” in that it invariably leaves out more detail about a system's structure than it includes (Williams et al., 2002). Misrepresented or omitted aspects of structure often lead to biased predictions and diminished management performance (Runge & Johnson, 2002).

One way to address this problem is to incorporate multiple prediction models directly into the decision‐making process and use management itself to learn the most effective way to predict the system's response. This is the purview of adaptive management, in which sequential decision‐making and learning can lead simultaneously to a reduction in uncertainty (i.e., better understanding) as well as improved management (Westgate et al., 2013). Following the seminal work by Holling (1978), Lee (1993) and Walters (1986), advances have been made in addressing conservation problems with different kinds of structural uncertainty (Fackler & Pacifici, 2014; O'Donnell et al., 2019; Williams, 2011a; Williams & Brown, 2016). However, implementation varies widely and inconsistently, as evidenced by the fact that there still are relatively few success stories (Westgate et al., 2013; Williams & Brown, 2014).

Here, we describe limited understanding about system structure and dynamics in terms of uncertainty as to which of several transition models is most appropriate, or which parameter value (e.g., survivorship and reproduction) is most appropriate (Figure 1). We account for this uncertainty with *K* models of system dynamics, along with model likelihoods $q\left(k\right)$ in a model state $q=\left\{q\left(k\right):k=1,,,\ldots ,,,K\right\}$ with $\sum_{k}q\left(k\right)=1.$

Structural uncertainty can be incorporated naturally into the basic Markovian framework described earlier for known decision processes. Thus, the same system states and actions are involved in system transitions, though multiple models $x^{\prime }=F_{k}\left(x,a,z\right)$ are used to produce model‐specific transitions $P_{k}\left(x^{\prime }|x,a\right)$. A new feature is the presence of a model state *q* and its propagation over time via Bayesian updating as the system state changes:

$$q^{\prime }\left(k\right)=\frac{q\left(k\right)P_{k}\left(x^{\prime }|x,a\right)}{P\left(x^{\prime }|x,a,q\right)},$$

where $P\left(x^{\prime }|x,a,q\right)=\sum_{k}q\left(k\right)P_{k}\left(x^{\prime }|x,a\right).$

Valuation for this problem uses model‐specific value functions

$$V_{\pi }^{k}\left(x\right)=R_{k}\left(a|x\right)+\lambda \sum_{x^{\prime }}P_{k}\left(x^{\prime }|x,a\right)V_{\pi }^{k}\left(x^{\prime }\right)$$

to get values that are averaged over the model state:

(3) $$\begin{array}{l} V_{\pi }\left(x,q\right)=\sum_{k}q\left(k\right)R_{k}\left(a|x\right)+\lambda \sum_{x^{\prime }}P\left(x^{\prime }|x,a,q\right)\sum_{k}q^{\prime }\left(k\right)V_{\pi }^{k}\left(x^{\prime }\right)\\ \;\;\;\;\;\;\;\;\;\;\;\;=R\left(a|x,q\right)+\lambda \sum_{x^{\prime }}P\left(x^{\prime }|x,a,q\right)V_{\pi }\left(x^{\prime },q^{\prime }\right) \end{array}$$

(Williams & Brown, 2022). Optimal valuation over potential policies π is given by

(4) $$V\left[x,q\right]=\max_{a}\left\{R\left(a|x,q\right)+\lambda \sum_{x^{\prime }}P\left(x^{\prime }|x,a,q\right)V\left[x^{\prime },q^{\prime }\right]\right\},$$

with an optimal policy consisting of the actions identified in value optimization: (Table 1).

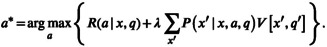




A comparison of the value function in Equation (2) for a known decision process,

$$V\left[x\right]=\max_{a}\left\{R\left(a|x\right)+\lambda \sum_{x^{\prime }}P\left(x^{\prime }|x,a\right)V\left[x^{\prime }\right]\right\},$$

and the value function in Equation (4) under structural uncertainty highlights key similarities and differences between known and structurally uncertain valuations. Thus, both variants of the Markovian framework include a combination of immediate and future returns; both involve a two‐part optimization, first optimizing the future conditional on the present and then optimizing the present given an optimal future; and both identify optimal strategies that are derived from their respective valuations. Unsurprisingly, strategy and valuation under structural uncertainty become the solution under structural certainty when a particular process is assumed to be known (when $q\left(k\right)=1$ for some *k*).

But there are also some key differences. For example, valuation under structural uncertainty averages returns and transitions over the model state, whereas averaging is unnecessary for valuation with a known decision process. State transitions under structural uncertainty involve the propagation of both system and model states, whereas only the system state is propagated for known processes. Perhaps the biggest operational difference concerns the nature of policies: policy π for a known process identifies actions to be taken for each system state at each decision point, whereas policy under structural uncertainty identifies actions to be taken for each *combination* of system and model states at each decision point. This makes a very big difference in finding solutions under structural uncertainty. Consider, for example, a problem with a univariate system state *x* (say, population size in some region) and four alternative models describing system change. When the model is assumed to be known, finding an optimal strategy is limited to a search for optimal values and actions over a one‐dimensional state space defined by *x*. On the other hand, structural uncertainty involves a search over a four‐dimensional space defined by *x* and *q*. Even if the likelihood values for $q\left(k\right)$ are assumed to be discrete, computing demands are much greater for the latter situation.

Strategy optimization in Equation (4) incorporates learning directly into the process of selecting actions, with future valuations that are based on updated model states as in $V\left[x^{\prime },q^{\prime }\right]$. It is the updating of model state from *q* to $q^{\prime }$ in the course of making decisions that represents learning. Decision‐making that simultaneously targets both learning and management in this way is often described as “active adaptive management” (Nichols & Williams, 2012).

An alternative form of decision‐making is “passive adaptive management,” which treats learning as a useful but unintended by‐product of management. The critical difference is that passive adaptive management uses future values $V\left[x^{\prime },q\right]$ in Equation (4) based on current rather than updated model states. This change means that decisions at each point are informed by future consequences only through the system response $x^{\prime }$, but not by learning per se; learning occurs after management action is taken, and *q* is updated to $q^{\prime }$. The restricted focus of decision‐making on system responses, with learning delayed until after a decision is selected, identifies this treatment as “passive” (Nichols & Williams, 2012). Though the strategy optimization in Equations (3) and (4) describes active adaptive management, both passive and active approaches to structural uncertainty are well represented in the ecological literature (Williams, 2011b).

The conservation literature cited at the beginning of this section clearly shows the usefulness of different models of system structure and different approaches to incorporating uncertainty about it into decision processes. It is increasingly important to include this uncertainty as a potential driver when making conservation decisions.

### 3.3 Dealing with partial observability

Another important conservation challenge concerns uncertainty about the status, or state, of an ecological system. The status of ecological systems is nearly always only partially observable, and variability is introduced when sampling produces state estimates that exhibit stochastic sampling variation. Failing to account for such variability can lead to poor management, as in the case of fishery harvest rules resulting in the collapse of the Argentine hake (*Merluccius hubbsi*) fishery (Memarzadeh et al., 2019), where the assumption of perfect observability in sampling led to misjudgment of population size and severe overfishing.

Partial observability can be addressed by including an additional uncertainty factor in dynamic decision‐making along with environmental variation (Figure 1). The explicit inclusion of uncertainty related to sampling (e.g., monitoring error) as a driver of optimal policy can significantly improve decision‐making, particularly for little‐known systems such as cryptic invasive forest insects (Fackler & Pacifici, 2014) or rare endangered species like the Sumatran tiger (*Panthera tigris sumatrae*; McDonald‐Madden, Chadès, et al., 2011). By now, there is a well‐developed theory and methodology to address limited or imperfect observability, mostly from the fields of robotics, artificial intelligence, and operations research (Poupart, 2005). The subject of accounting for partial observability has recently begun to appear in the ecological literature, for example, in overviews (Chadès et al., 2021; Williams & Brown, 2022) and case examples such as management of endangered seabirds (Tomberlin, 2010) and preservation of golden eagle (*Aquila chrysaetos*) nesting sites (Fackler et al., 2014). However, few conservationists know of its potential to improve ecological management.

As with structural uncertainty, the incorporation of partial observability builds directly on the basic Markovian framework for known, fully observed decision processes. A key difference is that sampling is used to produce an estimate that is not the same as the actual system state. Replacing known values of a system's state with distributions of possible states further complicates decision‐making, but potentially improves management effectiveness (Memarzadeh et al., 2019).

A formal expression utilizes the same system states *x* and actions *a* as in a fully observed decision process, except that the states are assumed to be unobservable. The same generic form $x^{\prime }=F\left(x,a,z\right)$ for the transition models is used, which again gives rise to Markovian transitions $P\left(x^{\prime }|x,a\right)$. Returns $r\left(a|x\right)$ also have the same form as in fully observed systems.

The new features are imperfect observability of *x* and the presence of sample observations that differ from, but are related to, the system's actual status. Observations before and after transitions are represented here by *o* and $o^{\prime }$, with an observation function $o^{\prime }=G\left(x^{\prime },,,a,,,\varepsilon \right)$ that produces random observations with a distribution $f\left(o^{\prime },|,x^{\prime },,,a\right).$ Since the actual state is unobservable, system status is represented by a probability distribution or “belief state” $b\left(x\right)$, which is propagated over time by combining monitoring data and prior belief state:

$$b^{\prime }\left(x^{\prime }\right)=\frac{f\left(o^{\prime },|,x^{\prime },,,a\right)\sum_{x}P\left(x^{\prime }|x,a\right)b\left(x\right)}{P\left(o^{\prime }|b,a\right)},$$

with

$$P\left(o^{\prime }|b,a\right)=\sum_{x^{\prime }}f\left(o^{\prime }|x^{\prime },a\right)\sum_{x}P\left(x^{\prime }|x,a\right)b\left(x\right).$$

Valuation is based on belief states rather than observable system states as in Equation (1). Thus, state‐specific returns $r\left(a|x\right)$ are averaged over the belief state to get $r\left(a|b\right)=\sum_{x}b\left(x\right)r\left(a|x\right)$ and accumulated into the value function

(5) $$\begin{array}{l} V_{\pi }\left(b\right)=E\left[\sum_{\tau =t}^{\infty }\lambda^{\tau -t}R\left(a_{\tau }|b_{\tau }\right)\left|b\right.\right]\\ \;\;\;\;\;\;\;\;\;=R\left(a|b\right)+\lambda \sum_{o^{\prime }}P\left(o^{\prime }|b,a\right)V_{\pi }\left(b^{\prime }\right). \end{array}$$

Optimal valuation with this function is given by

(6) $$V\left[b\right]=\max_{a}\left\{R\left(a|b\right)+\lambda \sum_{o^{\prime }}P\left(o^{\prime }|b,a\right)V\left[b^{\prime }\right]\right\},$$

with optimal policies consisting of the actions

$$a^{*}=\underset{a}{\arg\max}\left\{R\left(a|b\right)+\lambda \sum_{o^{\prime }}P\left(o^{\prime }|b,a\right)V\left[b^{\prime }\right]\right\}$$

identified in the value optimization (Table 1). An equivalent form of Equation (6) replaces the summation over observations $o^{\prime }$ by summation over belief states $b^{\prime }$, as in

(7) $$V\left[b\right]=\max_{a}\left\{R\left(a|b\right)+\lambda \sum_{b^{\prime }}P\left(b^{\prime }|b,a\right)V\left[b^{\prime }\right]\right\}$$

(Williams & Brown, 2022). A comparison of the value function in Equation (2) for known processes,

$$V\left[x\right]=\max_{a}\left\{R\left(a|x\right)+\lambda \sum_{x^{\prime }}P\left(x^{\prime }|x,a\right)V\left[x^{\prime }\right]\right\},$$

against the value function in Equation (7) for partially observed Markov processes not only highlights similarities between fully and partially observable dynamics but also points to some critical differences. For example, the value functions have quite similar forms, differing only in that state‐based returns and transitions are used when states are fully observed, whereas average returns and belief transitions are used under partial observability. A critical operational difference is that optimal values and actions for fully observable processes are defined over a discrete state space for *x*, whereas for partially observable processes they are defined over a continuous multidimensional belief space for *b*. This has a major impact on the approach to finding solutions (Chadès et al., 2021; Kaelbling et al., 1998; Williams & Brown, 2022).

A special case of partial observability that is especially relevant for conservation is *mixed observability*, which involves systems with some states that are observable and some that are not. For example, many conservation problems include features that are imperfectly observed (e.g., a rare population) and others that can be treated as fully observed (e.g., habitat condition).

Such “mixed observability” systems can be assessed by identifying states *x* for which observations coincide with system states $\left(o_{x}=x\right)$ and states *y* for which observations differ from system states $\left(o_{y}\neq y\right)$. Consider a system characterized by states $\left(x,y\right)$ with process transition probabilities $P\left(x^{\prime },y^{\prime }|x,y,a\right)$, and observations $\left(o_{x},o_{y}\right)$ with observation distribution $f\left(o_{x}^{\prime },o_{y}^{\prime }|x^{\prime },y^{\prime },a\right)$. The joint distribution for transitions and data is given by the product

$$P\left(x^{\prime },y^{\prime },o_{x}^{\prime },o_{y}^{\prime }|x,y,a\right)=P\left(x^{\prime },y^{\prime }|x,y,a\right)f\left(o_{x}^{\prime },o_{y}^{\prime }|x^{\prime },y^{\prime },a\right),$$

which simplifies to

$$P\left(x^{\prime },y^{\prime },o_{y}^{\prime }|x,y,a\right)=P\left(x^{\prime },y^{\prime }|x,y,a\right)f\left(o_{y}^{\prime }|x^{\prime },y^{\prime },a\right),$$

because *x* is observable. Bayesian belief updates for this situation are

$$\begin{array}{l} b^{\prime }\left(y^{\prime }\right)=P\left(y^{\prime }|x,x^{\prime },o_{y}^{\prime },b_{y},a\right)\\ \;\;\;\;\;\;\;\;\;=\frac{P\left(x^{\prime },y^{\prime },o_{y}^{\prime }|x,b_{y},a\right)\;}{P\left(x^{\prime },o_{y}^{\prime }|x,b_{y},a\right)}, \end{array}$$

where $b^{\prime }\left(y^{\prime }\right)$ is conditional on *x*, *a*, $x^{\prime }$ and $o^{\prime }$ (Williams & Brown, 2022).

Valuation for the mixed model is given by

$$\begin{array}{l} V_{\pi }\left(x,b_{y}\right)=\sum_{y}b\left(y\right)V_{\pi }\left(x,y\right)\\ \;\;\;\;\;\;\;\;\;\;\;\;\;\;\;=R\left(a|x,b_{y}\right)+\lambda \sum_{x^{\prime }}\sum_{o_{y}^{\prime }}\sum_{y^{\prime }}P\left(x^{\prime },y^{\prime },o_{y}^{\prime }|x,b_{y},a\right)V_{\pi }\left(x^{\prime },y^{\prime }\right) \end{array}$$

or, using the Bayesian posterior belief state $b_{y}^{\prime }$,

(8) $$V_{\pi }\left(x,b_{y}\right)=R\left(a|x,b_{y}\right)+\lambda \sum_{x^{\prime }}\sum_{o_{y}^{\prime }}P\left(x^{\prime },o_{y}^{\prime }|x,b_{y},a\right)V_{\pi }\left(x^{\prime },b_{y}^{\prime }\right).$$

With this expression, a mixed‐observability process can be seen as a natural extension of observable and unobservable processes (Table 1), in which returns, transitions, and values account for both types of system state. Removal of the unobservable state *y* reduces the mixed‐process valuation to that of a fully observable process. Alternatively, removal of the observable state *x* reduces the mixed‐process valuation to that of a partially observable process.

One important implication of the mixed model is the reduction of dimensionality for the belief space, which in turn reduces the computational burden in finding solutions with partially observable Markov decision process (POMDP) solvers (Nicol et al., 2015). Another implication is that adaptive decision‐making can be cast in a context of partial observability. To see how, let *y* represent an uncertain transition model with belief (model) state $b_{y}$ and restrict observations to the fully observable system state only $\left(o_{x}=x\right)$. The transition probabilities for the mixed model simplify to

$$P\left(x^{\prime },y^{\prime }|x,y,a\right)=P\left(x^{\prime }|x,y,a\right),$$

and the value function in Equation (8) becomes

(9) $$V_{\pi }\left(x,b_{y}\right)=R\left(a|x,b_{y}\right)+\lambda \sum_{x^{\prime }}P\left(x^{\prime }|x,b_{y},a\right)V_{\pi }\left(x^{\prime },b_{y}^{\prime }\right).$$

Then the optimal value function is

$$V\left[x,b_{y}\right]=\max_{a}\left\{R\left(a|x,b_{y}\right)+\lambda \sum_{x^{\prime }}P\left(x^{\prime }|x,a,b_{y}\right)V\left[x^{\prime },b_{y}^{\prime }\right]\right\},$$

which is just the value function

$$V\left[x,q\right]=\max_{a}\left\{R\left(a|x,q\right)+\lambda \sum_{x^{\prime }}P\left(x^{\prime }|x,a,q\right)V\left[x^{\prime },q^{\prime }\right]\right\}$$

in Equation (4) for the adaptive model with different notation. By framing adaptive management as a special case of mixed observability, this result allows the techniques and computer approaches to POMDPs to be brought to bear in finding solutions to adaptive management problems.

### 3.4 Dealing with nonstationarity

Finally, a challenge that confronts conservation everywhere is systemic or “nonstationary” change in the structure and functioning of biological systems over time. Nonstationarity is fast becoming a ubiquitous impediment to conservation, as unanticipated changes render past patterns no longer effective as predictors of the future (Milly et al., 2008). Climate change, widespread pollution, habitat fragmentation, disturbances and other factors can induce changes in ecological processes and thereby alter system dynamics over time. Failing to account for these impacts can lead to biased predictions and deleterious management strategies (Sutherland, 2006). Suggestions for incorporating nonstationarity in ecological management have included exploratory techniques, such as structured decision‐making (Martin et al., 2011; Nichols et al., 2011) and the use of scenario planning, as well as expert opinion and game theory (Sutherland, 2006).

Nonstationarity can be integrated formally into decision‐making by allowing for change in the system model over time as environmental and other factors change (Nicol et al., 2015). Thus, nonstationarity is characterized as a change from a model (or parameter) *y* to $y^{\prime }$, with transition probabilities $P\left(y^{\prime }|y\right).$ These probabilities serve as an added source of change along with state dynamics.

Here, we consider a nonstationary system that is only partially understood, with a joint distribution

$$\begin{array}{l} P\left(y,y^{\prime }\right)=P\left(y\right)P\left(y^{\prime }|y\right)\\ \;\;\;\;\;\;\;\;\;\;\;\;\;=q_{y}\left(y\right)q_{y^{\prime }|y}\left(y^{\prime }|y\right) \end{array}$$

of sequential models *y* and $y^{\prime }$, where $q_{y^{\prime }}\left(y^{\prime }\right)=\sum_{y}q_{y}\left(y\right)q_{y^{\prime }|y}\left(y^{\prime }|y\right)$. Structural uncertainties are represented by prior and posterior model states $q_{y}=\left[q_{y}\left(y\right)\right]$ and $q_{y^{\prime }}=\left[q_{y^{\prime }}\left(y^{\prime }\right)\right]$, respectively.

For this situation, valuation with resource state *x* and model *y* is given by

(10) $$V_{\pi }\left(x,y\right)=R_{y}\left(a|x\right)+\lambda \sum_{y^{\prime }}q_{y^{\prime }|y}\left(y^{\prime }|y\right)\sum_{x^{\prime }}P_{y^{\prime }}\left(x^{\prime }|x,a\right)V_{\pi }\left(x^{\prime },y^{\prime }\right),$$

where the immediate return $R_{y}\left(a|x\right)$ is based on model *y* and transitions $P_{y^{\prime }}\left(x^{\prime }|x,a\right)$ are based on model $y^{\prime }$. The value function in Equation (10) incorporates the sequence of an immediate return, followed by a stochastic transition from *y* to $y^{\prime }$, followed by a stochastic transition from *x* to $x^{\prime }$, with the summations essentially averaging the posterior values $V_{\pi }\left(x^{\prime },y^{\prime }\right)$ across the models $y^{\prime }$ and system states $x^{\prime }$.

Averaging over the prior model state $q_{y}$ then produces

(11) $$\begin{array}{ll} V_{\pi }\left(x,q_{y}\right)\; & =\sum_{y}q_{y}\left(y\right)V_{\pi }\left(x,y\right)\\ & =\sum_{y}q_{y}\left(y\right)R_{y}\left(a|x\right)+\lambda \sum_{y}q_{y}\left(y\right)\sum_{y^{\prime }}q_{y\prime |y}\left(y^{\prime }|y\right)\sum_{x^{\prime }}P_{y^{\prime }}\left(x^{\prime }|x,a\right)V_{\pi }\left(x^{\prime },y^{\prime }\right)\\ & =\sum_{y}q_{y}\left(y\right)R_{y}\left(a|x\right)+\lambda \sum_{y^{\prime }}\sum_{y}q_{y}\left(y\right)q_{y\prime |y}\left(y^{\prime }|y\right)\sum_{x^{\prime }}P_{y^{\prime }}\left(x^{\prime }|x,a\right)V_{\pi }\left(x^{\prime },y^{\prime }\right)\\ & =\sum_{y}q_{y}\left(y\right)R_{y}\left(a|x\right)+\lambda \sum_{y^{\prime }}q_{y^{\prime }}\left(y^{\prime }\right)\sum_{x^{\prime }}P_{y^{\prime }}\left(x^{\prime }|x,a\right)V_{\pi }\left(x^{\prime },y^{\prime }\right)\\ & =R\left(a|x,q_{y}\right)+\lambda \sum_{x^{\prime }}\sum_{y^{\prime }}q_{y^{\prime }}\left(y^{\prime }\right)P_{y^{\prime }}\left(x^{\prime }|x,a\right)V_{\pi }\left(x^{\prime },y^{\prime }\right)\\ & =R\left(a|x,q_{y}\right)+\lambda \sum_{x^{\prime }}\sum_{y^{\prime }}q_{y^{\prime }}^{\prime }\left(y^{\prime }\right)P\left(x^{\prime }|x,a,q_{y^{\prime }}\right)V_{\pi }\left(x^{\prime },y^{\prime }\right)\\ & =R\left(a|x,q_{y}\right)+\lambda \sum_{x^{\prime }}P\left(x^{\prime }|x,a,q_{y^{\prime }}\right)\sum_{y^{\prime }}q_{y^{\prime }}^{\prime }\left(y^{\prime }\right)V_{\pi }\left(x^{\prime },y^{\prime }\right)\\ & =R\left(a,x|q_{y}\right)+\lambda \sum_{x\prime }P\left(x^{\prime }|x,a,q_{y\prime }\right)V_{\pi }\left(x^{\prime },{q_{y\prime }}^{\prime }\right), \end{array}$$

where

$$q_{y^{\prime }}^{\prime }\left(y^{\prime }\right)P\left(x^{\prime }|x,a,q_{y'}\right)=q_{y^{\prime }}\left(y^{\prime }\right)P_{y^{\prime }}\left(x^{\prime }|x,a\right)$$

with $P\left(x^{\prime }|x,a,q_{y^{\prime }}\right)=\sum_{y^{\prime }}q_{y^{\prime }}\left(y^{\prime }\right)P_{y^{\prime }}\left(x^{\prime }|x,a\right).$

A comparison of the value function

$$V_{\pi }\left(x,q_{y}\right)=R\left(a|x,q_{y}\right)+\lambda \sum_{x^{\prime }}P\left(x^{\prime }|x,a,q_{y^{\prime }}\right)V_{\pi }\left(x^{\prime },q_{y^{\prime }}^{\prime }\right),$$

in Equation (11) under nonstationarity, against the form

(12) $$V_{\pi }\left(x,q_{y}\right)=R\left(a|x,q_{y}\right)+\lambda \sum_{x^{\prime }}P\left(x^{\prime }|x,q_{y},a\right)V_{\pi }\left(x^{\prime },q_{y}^{\prime }\right)$$

for stationarity from Equation (9), again not only highlights remarkable similarities in valuations for the two scenarios but also points to a critical difference: under stationarity, the state transitions $P\left(x^{\prime }|x,a,q_{y}\right)$ and valuations $V_{\pi }\left(x^{\prime },q_{y}^{\prime }\right)$ are based on the model *y*, whereas under nonstationarity the state transitions $P\left(x^{\prime }|x,a,q_{y^{\prime }}\right)$ and valuations $V_{\pi }\left(x^{\prime },q_{y^{\prime }}^{\prime }\right)$ are based on the posterior model $y^{\prime }$. The influence of model nonstationarity is clearly seen in the transition probabilities

$$P\left(x^{\prime }|x,a,q_{y^{\prime }}\right)=\sum_{y}q_{y}\left(y\right)q_{y^{\prime }|y}\left(y^{\prime }|y\right)P_{y^{\prime }}\left(x^{\prime }|x,a\right),$$

which include the model transition probabilities $q_{y^{\prime }|y}\left(y^{\prime }|y\right)$ that define nonstationarity. Their elimination (i.e., $P\left(y^{\prime }|y\right)=1\;\text{for}\;y^{\prime }=y)$ reduces nonstationary valuation in Equation (12) to the stationarity form in Equation (9).

This treatment of nonstationarity requires the specification of a Markov process $P\left(y^{\prime }|y\right)$ to represent the transition among models over time. Specification of such a process is necessarily speculative, in that the stochastic structure of change for, for example, climate change, is not known in advance. One way to deal with this uncertainty is to conduct value and policy assessments with different models of the process and investigate the range of policy implications under different scenarios of nonstationary change.

A different way to treat nonstationarity is to change the drivers rather than introducing new models, when nonstationarity is thought of as changes in the distribution of environmental driving variables (J. D. Nichols, personal communication). While this is certainly a valid and economical way of treating nonstationarity in many circumstances, a broader view allows for both perspectives. For example, a shift in driver variables giving rise to nonstationarity still requires the modeling of shift dynamics if future changes are also to be considered. Conversely, a change in a vital rate brought on by a single shift in an environmental driver can also be accommodated in our framework.

In many respects, nonstationarity is the most pressing of the conservation challenges we discuss in this paper, not least because of the accelerating rate of climate change and rapid pace of environmental alteration and degradation. With the incorporation of yet another source of uncertainty influencing system dynamics, nonstationarity also requires a more complex technical treatment.

## 4 DISCUSSION

In this paper, we have shown how four persistent and difficult problems in biological conservation—prediction of the future impacts of decisions; uncertainty about system structure; inability to observe biological systems fully; and nonstationary system dynamics—can be addressed within the same Markovian framework of controlled transitions over time. Modeling and estimation related to these problems have been major ongoing themes in the ecological and conservation literature. The framework presented in this paper can accommodate the necessary adjustments of state transition models, uncertainty metrics, and value functions to integrate the associated uncertainties into decision‐making.

These adjustments add considerable complexity, and incorporating them into ecological management has been uneven. Conservation has benefited from many decades of technical advances in dynamic optimization in systems analysis, operations research, and artificial intelligence, with widespread applications in finance, medicine, industrial logistics, and national defense (Bertsekas, 2017). There also has been considerable progress in the use of adaptive management to deal with structural uncertainty in ecological systems (Memarzadeh & Boettiger, 2018; Williams & Brown, 2016), though actual applications lag behind technical developments (Walters, 2007; Westgate et al., 2013).

On the other hand, advances in dealing with partial observability, especially in finding solutions for large and complex problems, have been addressed only relatively recently in the ecological literature (Chadès et al., 2021; Williams & Brown, 2022). Because methods for finding exact POMDP solutions scale poorly as problems increase in size and complexity, much of the work on partial observability has dealt with methods for finding solution approximations (Dujardin et al., 2017; Pineau et al., 2006; Poupart et al., 2011; Spaan & Vlassis, 2005). Finally, there is a wealth of information about the scale and scope of nonstationary dynamics, especially concerning climate change (IPCC, 2022), but few approaches exist for formally incorporating nonstationarity into ecological decision‐making (but see Martin et al., 2011; McDonald‐Madden, Runge, et al., 2011; Nichols et al., 2011; Nicol et al., 2015).

Several factors contribute to limited progress on these problems in ecology. These include the complexity of technical characterizations and notation; the inability to scale up exact methods for problems with large numbers of states and lengthy time horizons; the lack of a comparative context with which to recognize similarities and differences among the issues; and particularly, a lack of theoretically defensible frameworks and explanatory documentation that can aid in understanding issues and approaches.

Our development in this paper contributes to improved understanding of the four challenges in several ways. Our technical framework characterizing the conservation of dynamic systems, with states, state dynamics, controls, returns, and the other features highlighted, clarifies the linkages and similarities among the four challenges, while the uncertainty factors distinguish the models and motivate the differences in decision‐making with them. In addition, our technical development allows for the treatment of combined uncertainties over a broader range than is addressed in the current conservation literature. This aspect lays the groundwork for future progress on emerging issues, for example, incorporating nonstationarity simultaneously with structural uncertainty in dynamic decision‐making for systems affected by climate change.

The framework we have presented offers further opportunities to generalize the treatment of uncertainty and to focus on specific problems. For example, most problems in conservation simultaneously involve both partial observability and structural uncertainty. Integrating both sources of uncertainty into the same formulation could provide useful insights not seen with only a single uncertainty source (Williams, 2009). Expanding the framework to consider continuous as well as discrete state and action spaces would extend the range of applicability in ecological management, given that many problems are framed in terms of continuous variation over states and actions (Williams & Brown, 2022). An approach to structural uncertainty that allows research external to management to complement ongoing monitoring could expand the applicability of adaptive management (Williams, 2015) and address the relative value of information produced by monitoring and research (Williams & Brown, 2020). Other avenues of investigation include the integration of resilience into optimal stochastic decision‐making and valuation (Johnson et al., 2013); model‐free assessment in the spirit of reinforcement learning (Sutton & Barto, 2018); and perhaps most important to users, computing techniques to optimize the search for solutions.

In recent years, there has been greater recognition of the key role of ecological dynamics in conservation practice, along with rapid developments in modeling, assessment techniques, and computing power. These improvements promise to expand the useful range of the framework in this paper beyond the limited size and scope of problems to which it currently applies. Progress will build on contributions made to date, much as advances in the assessment of dynamic populations have continued to build on the important work of Jolly (1965) and Seber (1965) many years ago. A growing urgency to incorporate dynamics and associated uncertainties in decision‐making will likely lead to continuing methodological breakthroughs. It is important for ecological scientists and practitioners to engage in both the development and use of these advances, which will result in more effective conservation.

## AUTHOR CONTRIBUTIONS

**Byron K. Williams:** Conceptualization (lead); methodology (lead); writing – original draft (lead); writing – review and editing (equal). **Eleanor D. Brown:** Conceptualization (supporting); funding acquisition (lead); project administration (lead); writing – original draft (supporting); writing – review and editing (equal).

## CONFLICT OF INTEREST STATEMENT

The authors declare that they have no competing interests.

## Data Availability

No data were used in this study, and therefore, no data are available.
